# Draft Genome Sequence of Multidrug-Resistant *Pseudomonas aeruginosa* Strain VRFPA02, Isolated from a Septicemic Patient in India

**DOI:** 10.1128/genomeA.00425-13

**Published:** 2013-07-05

**Authors:** Jambulingam Malathi, Nandagopal Murugan, Vetrivel Umashankar, Radhakrishnan Bagyalakshmi, Hajib Narahari Rao Madhavan

**Affiliations:** Department of Microbiology, L & T Microbiology Research Centre, Vision Research Foundation, Sankara Nethralaya, Chennai, Indiaa; Centre for Bioinformatics, Vision Research Foundation, Sankara Nethralaya, Chennai, Indiab

## Abstract

Multidrug-resistant *Pseudomonas aeruginosa* strains, which are notable nosocomial pathogens, have greatly increased the mortality rate of septicemic patients due to treatment failure. Here, we report the draft genome sequence of *P. aeruginosa* strain VRFPA02, a human bloodstream isolate that has phenotypically proven to be resistant to a broad spectrum of antibiotics.

## GENOME ANNOUNCEMENT

*Pseudomonas aeruginosa* is an invading Gram-negative opportunistic pathogen that causes nosocomial infections and is responsible for 10% of all hospital-acquired infections (1). Infections caused by multidrug-resistant (MDR) *P. aeruginosa* are often severe and life-threatening and are difficult to treat due to their narrow range of susceptibility to antimicrobial agents. The constant emergence of antibiotic resistance during therapy results in adverse therapeutic outcomes (2). Since *P. aeruginosa* has innate potential to develop resistance to virtually any antibiotic to which it is exposed, due to the acquisition of multiple resistance mechanisms, it becomes an MDR strain. The resistance mechanism of *P. aeruginosa* is exhibited through the production of beta-lactamases, and various efflux pump systems present are also capable of effectively purging most of the antibiotics from the intracellular environment. Hence, understanding the intrinsic and extrinsic mechanisms of antimicrobial resistance in *P. aeruginosa* may aid in exploring novel drug targets to combat nosocomial infections.

We announce the draft genome of the MDR *P. aeruginosa* VRFPA02 strain, isolated from a human blood specimen from the L & T Microbiology Research Centre, Sankara Nethralaya, Chennai, India. The isolated strain was found to be resistant to the broad-spectrum antibiotics, namely cephalosporins, aminoglycosides, fluoroquinolones, and carbapenems (except for imipenem). Furthermore, the production of metallo-beta-lactamases (MBL) by the strain was confirmed by the combined-disk method (3). Hence, to deduce the genomic insights of drug resistance in this isolate (VRFPA02 strain), whole-genome sequencing was performed using Ion Torrent (PGM) sequencer with 200-bp read chemistry (Life Technologies). Standard protocols as per the manufacturer’s instructions were followed during the sequencing process. Genomic DNA from VRFPA02 strain was isolated from an overnight culture using the DNeasy miniprep kit (Qiagen, Hilden, Germany). Initially, identification and confirmation of the strain to the species level were carried out by 16S rRNA gene sequencing. The genomic library was constructed using 10 µg of genomic DNA from VRFPA02 through enzymatic digestion to generate an average of 200-bp DNA fragments. The resultant libraries were diluted to the recommended concentrations determined by a high-sensitivity DNA assay (Agilent Technologies) and were further used as templates for emulsion PCR (emPCR). Finally, the enriched emPCR product was loaded onto a 316 chip and sequenced using the Ion PGM 200 sequencing kit. After the sequencing run, the generated data were filtered with a Phred score cutoff of ≤20. The filtered sequences were *de novo* assembled using CLC Genomics Workbench software version 5.5.1 (CLC bio, Germantown, MD), wherein 186 contigs with 50× genome coverage were obtained. The sequenced product ranged from 2,000 to 102,027 bp in size. The assembled data were subjected to RAST annotation, and the genome size was found to be 6,474,120 bp, comprising 6,500 protein-coding genes and 63 RNA-coding genes (4). *P. aeruginosa* genomic island 4 (PAGI-4), PAGI-5, PAGI-9, PAGI-10, and PAGI-11, which were not detected in *P. aeruginosa* strain PAO1, were present in VRFPA02 (5). Sequences were annotated using the NCBI Prokaryotic Genomes Automatic Annotation Pipeline (PGAAP) (http://www.ncbi.nlm.nih.gov/genomes/static/Pipeline.html).

A more-detailed comparative analysis of this genome with those of other multidrug-resistant *P. aeruginosa* strains will provide further insight into the specific properties related to MDR.

### Nucleotide sequence accession numbers.

This whole-genome shotgun project of *P. aeruginosa* strain VRFPA02 has been deposited at DDBJ/EMBL/GenBank under the accession no. AQHM00000000. The version described in this paper is the first version, AQHM00000000.1.
